# A Newly Developed TGF-Β-Responsive CAR T Cell for Enhanced Proliferation and Cytokine Secretion

**DOI:** 10.34172/apb.025.45483

**Published:** 2025-08-30

**Authors:** Shafieeh Mansoori, Mohammad Ali Shokrgozar, Monireh Gholizadeh, Shahriyar Abdoli, Soheila Ajdary, Zahra Sharifzadeh

**Affiliations:** ^1^Department of Immunology, Pasteur Institute of Iran, Tehran, Iran; ^2^Student Research Committee, Pasteur Institute of Iran, Tehran, Iran; ^3^National Cell Bank of Iran, Pasteur Institute of Iran, Tehran, Iran; ^4^Department of Medical Biotechnology, Faculty of Advanced Medical Sciences, Tabriz University of Medical Sciences, Tabriz, Iran; ^5^School of Advanced Medical Technologies, Golestan University of Medical Sciences, Gorgan, Iran

**Keywords:** Chimeric antigen receptor therapy, Immunosuppression, Adoptive cellular immunotherapy, Solid tumor, Transforming growth factor beta, Tumor microenvironment

## Abstract

**Purpose:**

Chimeric antigen receptor (CAR) T cell therapy has emerged as a promising cancer treatment. Nevertheless, the tumor microenvironment (TME) of solid tumors provides substantial challenges to CAR T cell efficacy. Tumor growth factor-beta (TGF-β), a potent immunosuppressive cytokine in the TME, impedes T cell activation, proliferation, and cytotoxicity, diminishing the anti-tumor potency of CAR T cells. This study investigates whether TGF-βRII CAR T cells can overcome these barriers and remain functional in TGF-β-rich environments.

**Methods:**

We developed a novel TGF-βRII CAR T cell (TGF-βRII-CD28CD3z) and a dominant-negative TGF-β receptor (dnTβRII) T cell utilizing Jurkat cells. Transduction efficiency and surface expression were confirmed using flow cytometry. T cell activation and proliferation were assessed by CD69 and Ki-67 expression, respectively. IL-2 and IFN-γ secretion were quantified using ELISA kits.

**Results:**

Flow cytometry confirmed the successful cell surface expression of the designed receptors: 62% and 24% for TGF-βRII CAR and dnTβRII, respectively. TGF-βRII CAR T cells were markedly activated in a dose-dependent manner, with optimal responses at 10 ng/mL TGF-β. The Ki-67 expression of CAR T cells, used as a proliferation marker, increased 1.21-fold (from 79.5% to 96%) upon exposure to 10 ng/mL TGF-β. At 5 ng/mL TGF-β, the cells’ proliferation was maintained at a 1.04-fold increase. Cytokine analysis revealed a 1.9-fold increase in IL-2 (130±4 pg/mL) and a 2.7-fold increase in IFN-γ (146±21.9 pg/mL) secretion at 10 ng/mL TGF-β. Additionally, at 5 ng/mL TGF-β, IL-2 secretion increased 1.6-fold (110±10.7 pg/mL), and IFN-γ secretion increased 1.7-fold (94.3±10.2 pg/mL). In contrast, dnTβRII T cells also produced IL-2 (95 pg/mL±22, 2.7-fold increase) but failed to sustain proliferation or IFN-γ production at 10 ng/mL TGF-β.

**Conclusion:**

Our findings demonstrate that the TGF-βRII CAR T cells not only resist TGF-β-mediated suppression but also promote activation, proliferation, and cytokine release in the presence of TGF-β. This underscores their therapeutic potential as an innovative approach to overcome TGF-β-driven immunosuppression and improve the CAR T cell therapy efficacy in solid tumors.

## Introduction

 Chimeric antigen receptor (CAR) T cell therapy has emerged as a promising approach in oncology, particularly for treating hematological malignancies. This innovative strategy utilizes genetically engineered T cells to specifically target and eliminate tumor cells. Nevertheless, some challenges remain to be addressed, particularly in the solid tumor environment, where the efficacy of CAR T cells is hindered by the highly immunosuppressive tumor microenvironment (TME). Approved CAR T-cells have shown sustained clinical responses in patients with hematological malignancies; however, some of these patients experienced relapse. Additionally, there are reports of CAR T-cell non-responders. A possible explanation for these failures could be the transduction of inhibitory signals to CAR T cells, rendering them ineffective. Among the various immunosuppressive factors, transforming growth factor-beta (TGF-β) stands out as a potent inhibitor of immune responses, hindering the anti-tumor activity of CAR T cells.^1,2^

 TGF-β, a cytokine with a dual role in cancer biology, can regulate cell growth, differentiation, angiogenesis, invasion, and immune responses.^3^ It is a key player in tumor progression, acting as a master regulator of immune responses within the TME.^4^ Its pleiotropic effects include the suppression of immune surveillance, the promotion of immune tolerance, and the facilitation of tumor immune evasion.^5^ There are three known isoforms of TGF-β in mammalian cells: TGF-β1, TGF-β2, and TGF-β3. These isoforms are produced by various cell types, including normal, tumor, and stromal cells,^6^ primarily as inactive homodimers or latent complexes.^7^ Among them, TGF-β1 is the most widely expressed isoform.^8^ Despite the widespread production of TGF-β, its activation is limited to areas like the TME, which contains integrins and metalloproteinases in its extracellular matrix.^9^ Only the active form of TGF-β can bind to the TGF-β receptor II (TβRII) and perform its biological functions in immune cells.^6^ One mechanism by which TGF-β exerts its immunosuppressive effects is through directly impairing the anti-tumor functions of T cells, including their activation, proliferation, and cytotoxicity.^10,11^ Additionally, TGF-β has been found to repress the secretion of IFN-γ and IL-2 in activated T cells.^12^ This poses a major challenge for CAR T cell therapy, which relies on the activation and effector functions of T cells to target and eliminate tumor cells. To address these challenges, previous studies have explored strategies to restore anti-tumor activity in T cells -by eliminating the inhibitory effects of TGF-β. These methods include the use of small molecules that block the TGF-β signaling pathway, anti-TGF-β antibodies, TGF-β traps, antisense oligonucleotides targeting TGF-β, and TGF-β receptor inhibitors.^13,14^ However, these systemic strategies block TGF-β signaling in all cell types, including tumor and normal cells, which could interfere with physiological TGF-β functions that are vital for tissue homeostasis. Other approaches have focused on engineering T cells to specially enhance their resistance to TGF-β-mediated inhibition. These include the incorporation of dominant negative TGF-β receptors (dnTβRs) into the T cells/CAR construct, which sequester TGF-β and prevent its binding to endogenous receptors on T cells.^15-19^ Additionally, deleting the endogenous TGFβRII in CAR T cells by CRISPR/Cas9 systems can disrupt TGF-β signaling pathways, making CAR T cells resistant to its inhibitory effects.^20,21^ Compared to CAR T cells with TGFβRII-knockout, dnTβRII T cells exhibit greater proliferative capacity despite persistent TGF-β signaling through endogenous TGF-β receptors.^17^ However, dnTβRII T cells are unable to recruit other immune cells such as NK cells, CD8 + T cells, and dendritic cells due to their lack of pro-inflammatory cytokine secretion. Given the limited effectiveness of current methods targeting TGF-β signaling, there is a compelling necessity to develop new and improved platforms. In more innovative strategies, researchers attempt to design CAR T cells that are not only resistant to TGF-β but also become activated upon TGF-β exposure to enhance anti-tumor responses.^22-24^ These CAR T cells express a chimeric receptor composed of an ectodomain, a transmembrane domain, and an endodomain, which is the functional signaling portion of the receptor. The signaling endodomain typically includes the CD3ζ chain paired with either the CD28 or 4-1BB co-stimulatory domains, each triggering distinct intracellular signaling pathways upon ligand binding through the ectodomain. CD28 signaling induces rapid and strong effector functions but is associated with shorter CAR T cell persistence. In contrast, 4-1BB signaling promotes sustained, memory-like CAR T responses with enhanced longevity.^25-27^ Notably, CD28-based CAR T cells exhibit greater resistance to TGF-β-mediated inhibition of T cell proliferation by maintaining IL-2 signaling, resulting in more potent effector activity.^28^ Besides, CD28-CD3ζ CAR T cells sustain significantly higher steady-state levels of IL-2 and IFN-γ.^28,29^ Given these properties, we selected the CD28-CD3ζ to drive faster and stronger responses within the suppressive TGF-β–rich TME.

 In the present study, we developed novel TGF-βRII CAR T cells in which TGF-β receptor II (TβRII) is fused to the CD28-CD3ζ signaling endodomain. These CAR T cells are expected to become activated upon binding to TGF-β via their stimulatory endodomain. Therefore, their activation was evaluated by measuring proliferative responses and the release of pro-inflammatory cytokines in the presence of TGF-β.

## Materials and Methods

###  Cell lines and culture conditions

 Lenti-X293T, HEK-293T, and Jurkat cell lines were purchased from the National Cell Bank of Iran (NCBI), Pasteur Institute of Iran. Lenti-X293T and HEK-293T cells were maintained in Dulbecco-modified Eagle medium (DMEM, Sigma-Aldrich, USA) supplemented with 10% heat-inactivated fetal bovine serum (FBS, Bioserea, France), 2 mM L-glutamine, 100 units/mL penicillin, and 100 ug/mL streptomycin (Gibco, Carlsbad, CA). The Jurkat cell line was maintained in complete RPMI containing RPMI 1640 (Sigma-Aldrich, USA), 10% heat-inactivated FBS, 100 units/mL penicillin, and 100 ug/mL streptomycin, with or without TGF-β1 (100-21, PeproTech). All cells were incubated at 37°C in a humidified incubator with 5% CO_2_.

###  DNA constructs

 A second-generation TGF-βRII CAR was synthesized by Biomatik Company (Cambridge, Canada), incorporating the GM-CSF signal peptide, a c-Myc tag for confirming surface expression of CAR, the extracellular domain (ECD) of TGF-βRII (NM003242, variant B), the CD28 transmembrane (TM) domain, and the CD28 costimulatory and CD3ζ signaling domains. The TGF-βRII CAR construct was cloned into the transfer lentiviral vector pCDH under the control of the CMV promoter and included GFP as a surrogate marker. The dnTβRII construct was generated by replacing the CD28/ CD3ζ signaling domains of the TGF-βRII CAR with mCherry marker while GFP remained. The pCDH, as the backbone of TGF-βRII CAR and dnTβRII, is an HIV-1 derived second-generation self-inactivating lentiviral vector containing a multiple cloning site downstream of the CMV promoter, bacterial replication elements, important regulatory elements, and GFP as a surrogate marker.

###  Lentivirus production and titration

 Lentiviral vectors encoding TGF-βRII CAR or dnTβRII constructions were generated by co-transfecting cells with three plasmids: the packaging plasmids psPAX2 and pMD2.G, along with transfer plasmid pCDH at a ratio of 1:2.5:2.5. These plasmids were delivered into lenti-x293T packaging cells using polyethyleneimine 25000 (PEI 25000, Sigma) as the transfection reagent. At the end of the 72-hour of co-transfection, the efficiency of the transfection was quantified via flow cytometry.

 After transfection, the supernatant containing lentiviral particles was collected every 8 to 12 hours for four days and concentrated by ultracentrifugation at 50,000 × g for 7 h at 4 °C. For the confirmation of the lentiviral production, HEK-293T cells were transduced with the concentrated lentiviral vector along with 8 μg/mL of polybrene (H9268, sigma), as described previously.^30^ Briefly, 1 × 10^5^ cells/well were transduced with the vector volumes of 1 μL, 10^-1^ μL, 10^-2^ μL, 10^-3^ μL, and 10^-4^ μL, respectively, in a 12-well plate. Three days later, flow cytometry was done to examine GFP expression and determine the transduction efficiency. The viral titers were figured out from the dilutions with less than 20% GFP-positive cells via the following formula:


$$Viral\;titer\;\left(\frac{TU}{ml}\right)=\frac{\text{number of Hek}293\text{T }\left(\text{count at transduction day}\right)\text{x }\frac{\%GFP+cells}{100}}{\text{volume of the virus }\left(\text{ml}\right)}$$

###  Generation of stable engineered T cells

 In a 24-well plate, 1 × 10^5^ Jurkat cells/well were transduced by 8 μg/mL polybrene and TGF-βRII/or dnTβRII concentrated virus at an MOI > 10, and incubated at 37 °C. The required viral volume for a certain MOI was estimated using the following equation:


$$\text{The required viral volume }\left(\text{mL}\right)=\frac{\text{total cell number}\;\times MOI}{\text{virus titer }\left(\frac{\text{TU}}{\text{mL}}\right)}$$

After three days, the transduction rate was confirmed by flow cytometry. Transduced cells were selected with 1 μg/mL puromycin over a two-week period and subsequently analyzed by flow cytometry for GFP expression. To generate mock cells as a control, Jurkat cells were transduced with the empty pCDH vector (without any insert). All subsequent assays utilized engineered cells with a GFP expression ≥ 98%. Throughout all the tests, puromycin was excluded from the culture media. Cell-surface expressions of dnTβRII and TGF-βRII CAR T cells were measured using a PerCP-Cy5.5-labeled anti-c-Myc antibody (SC40, santa Cruz).

###  Activation assay

 The dnTβRII, TGF-βRII CAR T cells, and mock cells were seeded in 96-well U-bottom plates at 3 × 10^4^ cells/well in the presence of the varying concentrations of TGF-β (0, 5, 10 and 50 ng/mL). After 18 h, the cell activation rate was assessed by collecting the cells and analyzing their surface expression of CD69 using flow cytometry. For cell preparation, cells were washed twice with PBS and then incubated with the PE-anti-human CD69 antibody (BioLegend, USA) for 40 min in the dark at 4 °C. After incubation, the cells were washed again and analyzed by flow cytometry (Partec PAS-III) using FlowJo software (V10.6.2).

###  Proliferation assay

 To evaluate proliferation, engineered cells were seeded at a density of 3 × 10^4^ cells/well, were treated with 0, 5, and 10 ng/mL TGF-β, and incubated for three days at 37 °C in a humidified incubator with 5% CO_2_. The cells were fixed with 2% formaldehyde and permeabilized with 70% ice-cold ethanol, followed by staining with PE-conjugated anti-human Ki-67 (BioLegend, USA) antibody. Proliferation was then assessed by flow cytometry analysis.

###  Cytokine assay

 Cytokine release was measured using human IL-2 (BioLegend, USA) and human IFN-γ (Karmania Pars Gene, Iran) Elisa kits. The cells were plated in 96-well U-bottom plates at a density of 3 x 10^4^ cells/well and treated with or without TGF-β (10 or 0 ng/mL). Forty-eight hours post-stimulation, the supernatant was collected, centrifuged at 300 × g for 5 min at 4 °C, and stored at -80 °C until use.

###  Statistics

 Data analysis was performed using GraphPad Prism 8 software (GraphPad Software, V.10.2.3). One-way or two-way ANOVA with Tukey’s post hoc test was used for comparative analysis between three or more groups. *P* values < 0.05 were regarded as statistically significant.

## Results

###  Lentiviruses are produced with a high functional titer

**Figure 1:**
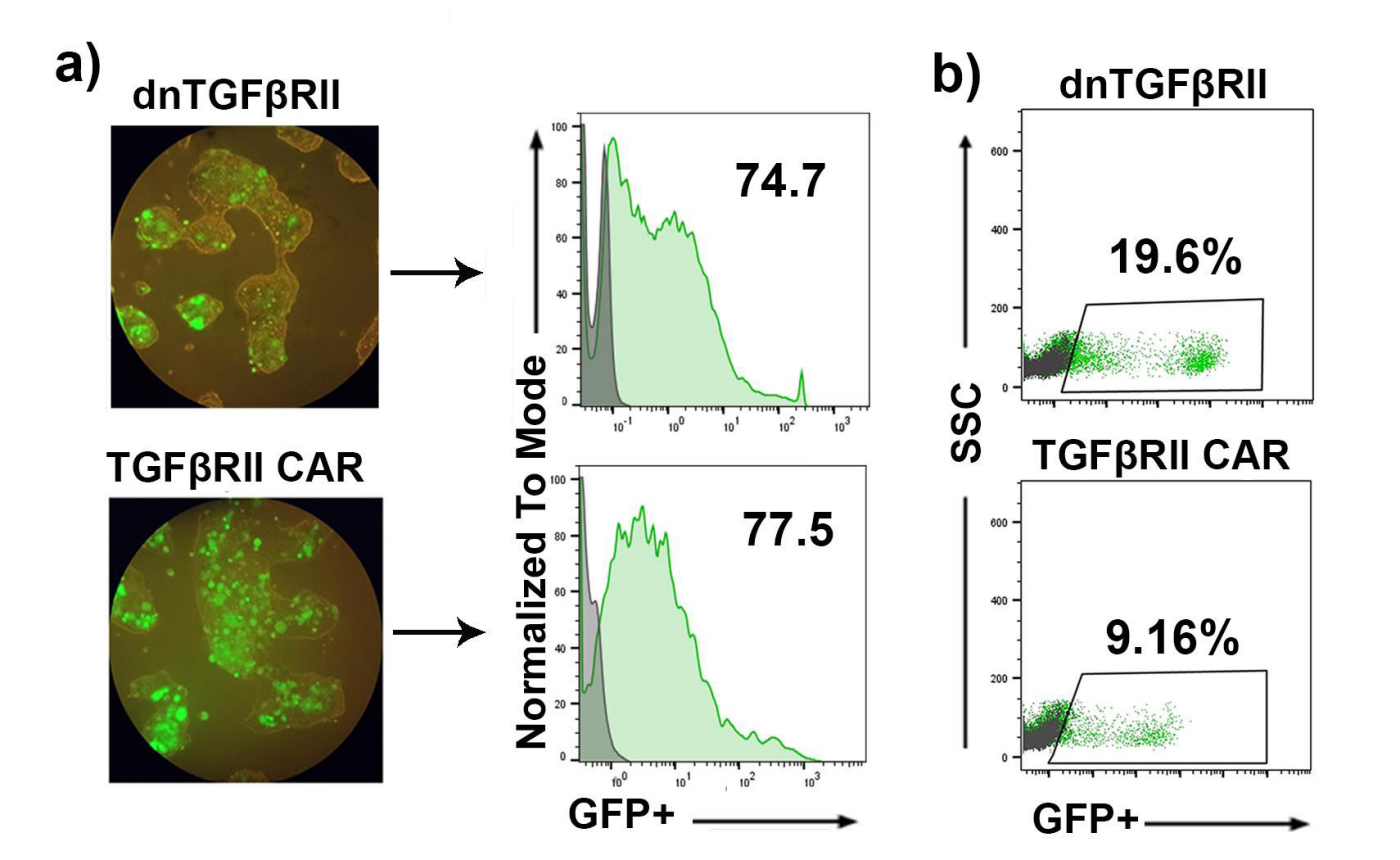
Transfection rate and lentiviral titration of dnTβRII and TGF-βRII CAR. a) Fluorescence microscopy photographs and Flow cytometry analysis of HEK-293T cells transfected with dnTβRII and TGF-βRII CAR vectors. The GFP-positive cells indicate successful transfection. b) Flow cytometry analysis of the lentiviral titer through transducing HEK-293T cells with lentiviral vectors expressing dnTβRII and TGF-βRII CAR. The GFP-positive cells indicate successful transduction. Control cells (gray); transfected/or transduced cells (green)

 Transfection efficacy was assessed via fluorescence microscopy and flow cytometry, both of which confirmed the presence of green-fluorescent cells (Figure 1a, b). Moreover, flow cytometry analysis revealed transfection rates of 75% and 77% for dnTβRII and TGF-βRII TGF-β CAR lentiviral vectors, respectively, confirming efficient vector delivery (Figure 1b).

 The supernatant from transfected HEK-293T cells was concentrated by ultracentrifugation. To quantify lentiviral production and calculate the functional titer, HEK-293T cells were transduced with the concentrated virus, and thepercentage ofGFP-positive cells was analyzed by flow cytometry three days post-transduction. As illustrated in Figure 1c, transduction with dnTβRII yielded 19.6% GFP-positive cells, compared to 9.16% with TGF-βRII CAR. These results were obtained by transducing 1.25 × 10^5^ and 1.5 × 10^5^ cells, respectively, with 1 μL of the dnTβRII and TGF-βRII CAR viruses. Based on these data, the lentiviral titers were calculated as 2.4 × 10^7^ TU/mL for TGF-βRII CAR and 1.37 × 10^7^ TU/mL for dnTβRII. Additionally, mock lentiviruses were produced in a similar manner, yielding a transfection efficiency of 93% (Supplementary file, Figures S1a and S1b).

 These findings indicate that lentiviruses expressing TGF-βRII CAR and dnTβRII constructs can efficiently produce a functional viral titer.

###  TGF-βRII CAR is efficiently expressed on transduced T cells

**Figure 2:**
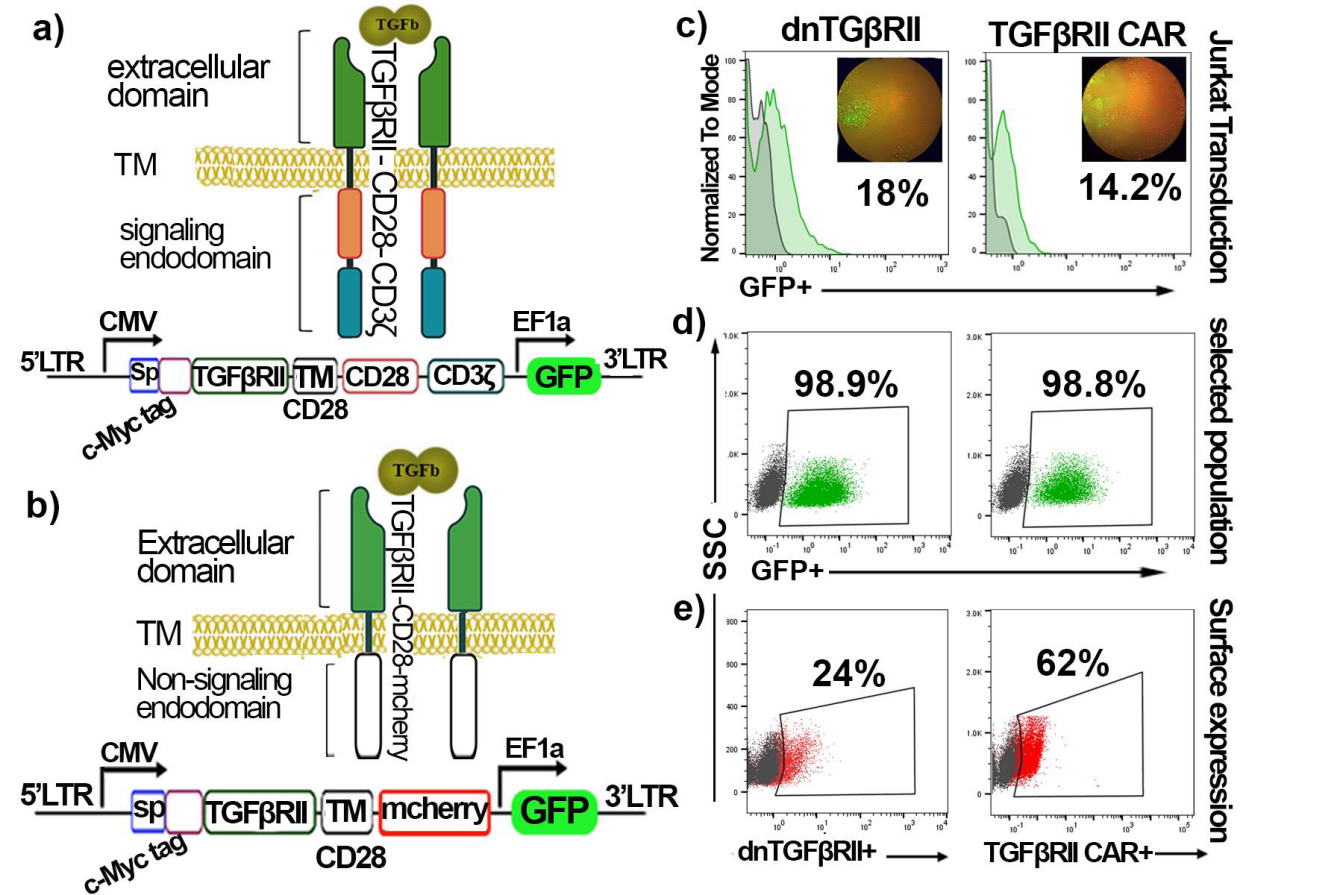
Schematic of the designed Constructions and their characterization. **a) **The TGF-βRII CAR construct consists of a signal peptide, a cmyc-tagged TGF-βRII extracellular domain (which binds to TGF-β), fused to the CD28 Transmembrane (TM) domain, followed by the CD28 co-stimulatory domain and the CD3ζ signaling domain. **b)** The dnTβRII was made by linking a signal peptide, c-Myc-tagged TGF-βRII extracellular domain to mCherry marker as an endodomain through CD28 TM. *The GFP marker is present in the pCDH lentiviral vector. **c)** Transduction rate (GFP + cells) of Jurkat cells with dnTβRII and TGF-βRII CAR. **d)** Flow cytometry analysis of dnTβRII and TGF-βRII CAR Jurkat cells selected with puromycin after two weeks. **e)** The surface expression of dnTβRII and TGF-βRII CAR on the Jurkat cells. Control cells (gray); transduced cells (green); c-Myc expressing cells (red). PerCP-Cy5.5-labeled anti-c-Myc antibody is used for the surface expression assessment

 To generate TGF-βRII CAR T cells and dnTβRII T cells, Jurkat cells were separately transduced with lentivirus expressing the TGF-βRII CAR (Figure 2a) and dnTβRII (Figure 2b) constructs, respectively. The transduction rate was measured by detecting the percentage of GFP^+^cells using flow cytometry. The results showed that 14.2% and 18% of cells were transduced with the TGF-βRII CAR and dnTβRII viruses, respectively (Figure 2c). In contrast, mock T cells, serving as a control, exhibited a transduction efficacy of 69.4% (Figure S1c). To isolate stable transduced cells and eliminate non-transduced cells, the cell cultures were treated with puromycin for two weeks. Following this selection process, the percentage of GFP-positive cells, along with the surface expression levels of TGF-βRII CAR and dnTβRII, were assessed. As demonstrated in Figure 2d, TGF-βRII CAR T cells and dnTβRII T cells achieved approximately 98% GFP positivity. In comparison, mock T cells exhibited a screening efficiency of ≥ 99% (Figure S1d).

 Furthermore, flow cytometry analysis revealed that the TGF-βRII CAR and dnTβRII were expressed on the surface of transduced T cells at rates of 62% and 24%, respectively. These results indicate that both the transduction and selection processes were effective in enriching cells that express the desired constructs and that TGF-βRII CAR and dnTβRII could be efficiently expressed on the surface of the T cells.

###  TGF-βRII CAR T cells can be activated in the presence of TGF-β

**Figure 3:**
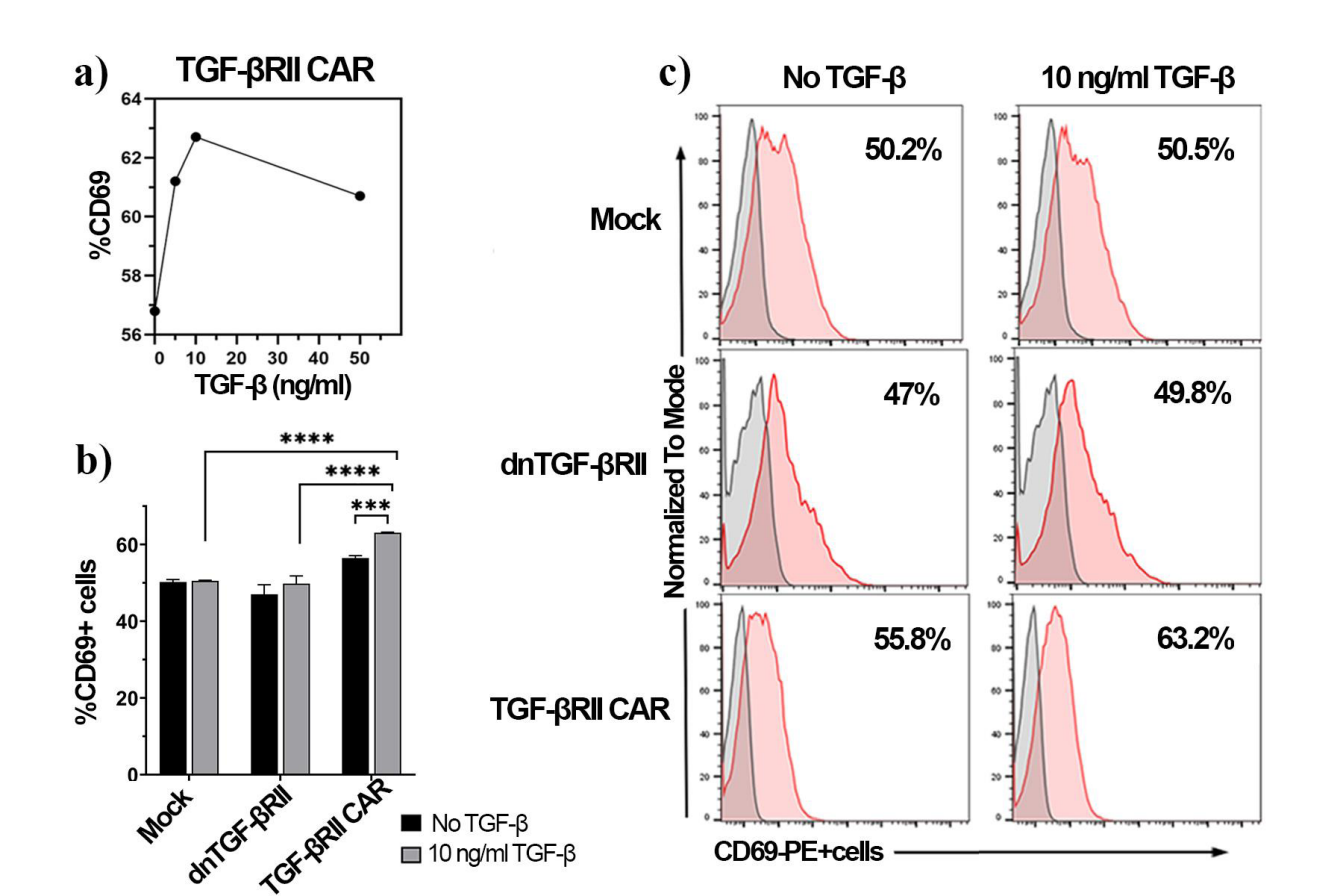
Activation of TGF-βRII CAR T cells and control cells in response to TGF-β stimulation after 18 h, assessed by CD69 expression. **(a)** Dose-response curve of CD69 expression in TGF-βRII CAR T cells after stimulation with varying concentrations of TGF-β (0, 5, 10 and 50 ng/mL), **(b)** Comparison of CD69 expression in mock, dnTβRII, and TGF-βRII CAR T cells following stimulation with no TGF-β (black bars) and 10 ng/mL TGF-β (gray bars), **(c)** Flow cytometry histograms showing CD69 expression profiles for mock, dnTβRII, and TGF-βRII CAR T cells, stimulated with or without 10 ng/mL TGF-β. Percentages represent the proportion of CD69-positive cells (red); the unstained cells used as the control (gray). Statistical analysis was performed using a two-way ANOVA test, and significance is indicated by *P* < 0.001 (***) and *P* < 0.0001 (****). Data represent the mean ± SD of three technical replications per assay. CD69 expression was measured by PE-anti-human CD69 antibody

 Activation and proliferation assays were performed to evaluate the responsiveness of TGF-βRII CAR T cells to TGF-β stimulation. At first, TGF-βRII CAR T cells were exposed to various concentrations of TGF-β (0, 5, 10, and 50 ng/mL). After 18 hours, their activation profiles were assessed by surface CD69 expression detection. The TGF-βRII CAR T cells exhibited a dose-dependent response to TGF-β stimulation (Figure 3a), with the percentage of CD69 + cells increasing from 56.8% (0 ng/mL TGF-β) to 61.2% (5 ng/mL) and 62.7% (10 ng/mL). However, at 50 ng/mL, the percentage of CD69 + cells did not increase further beyond the levels observed at 10 ng/mL stimulation (Figure 3a). These findings suggested that TGF-βRII CAR T cells activate in the presence of TGF-β, with optimal activation observed at 10 ng/mL TGF-β. Therefore, 10 ng/mL TGF-β was used to evaluate the function of mock and dnTβRII T cells.

 Next, the activation of TGF-βRII CAR T cells after 18 hours of stimulation with 10 ng/mL TGF-β was compared to mock-transduced and dnTβRII T cells. As illustrated in Figures 3b and c, both mock (50.2% ± 0.64 vs 50.5% ± 0.18) and dnTβRII T (47% ± 2.5 vs 49.8% ± 2) cells showed no significant differences in CD69 expression with or without TGF-β, demonstrating their lack of responsiveness. In contrast, TGF-βRII cells exhibited increased CD69 expression (63% ± 0.15) under TGF-β stimulation, markedly surpassing the levels observed in both control cell types (*p*< 0.0001) (Figure 3b).TGF-βRII cells demonstrated considerable activation compared to the baseline levels (56.4% ± 0.65, *P* = 0.0008), confirming the TGF-βRII CAR’s capacity to mediate T-cell activation.

 Together, these results demonstrated that the TGF-βRII CAR T cells were able to recognize and respond to TGF-β stimulation, unlike the mock and dnTβRII T cells, which do not exhibit these responses.

###  TGF-βRII CAR T cells can proliferate in the presence of TGF-β

**Figure 4:**
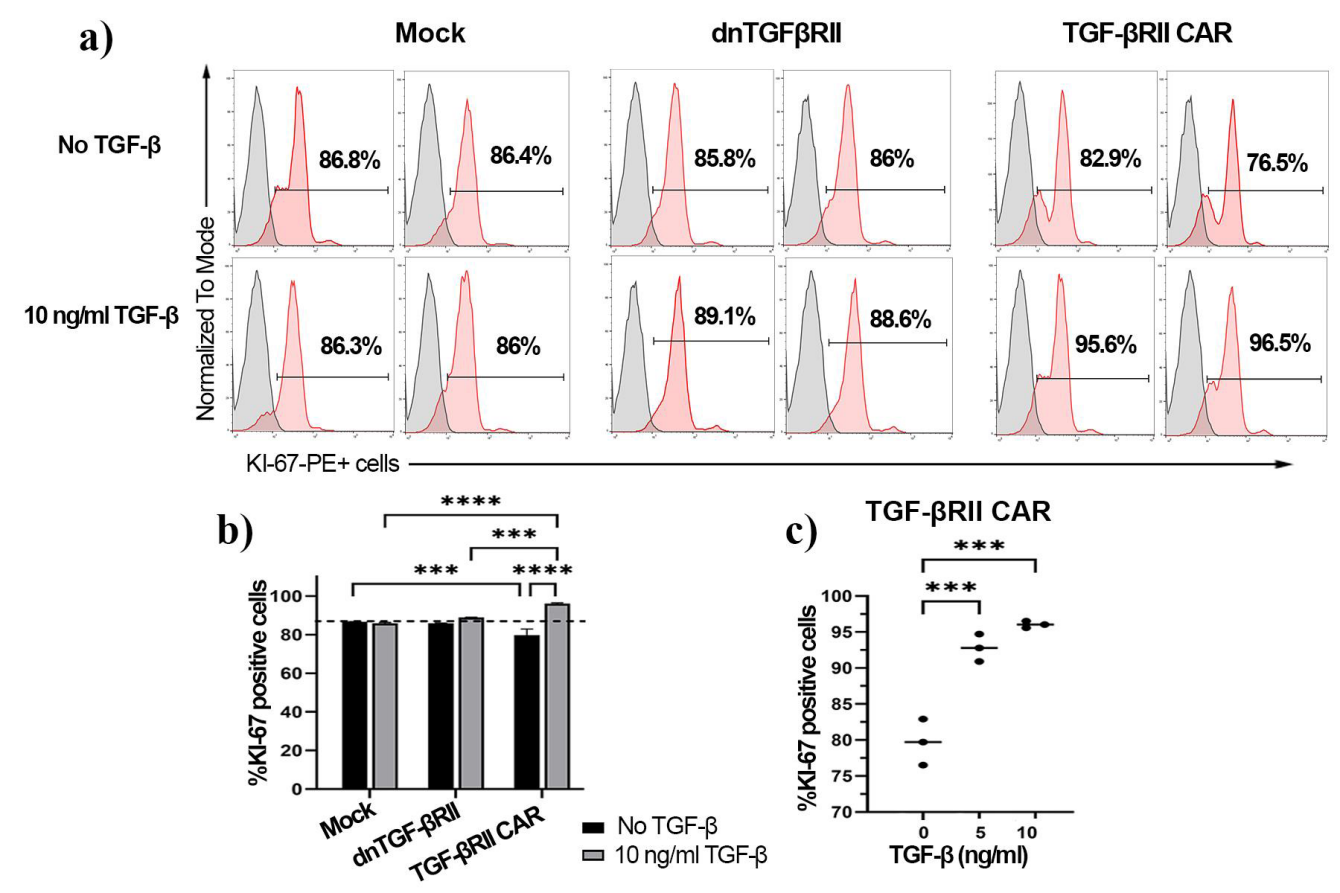
Proliferation analysis of TGF-βRII CAR T cells compared to mock and dnTβRII T cells in response to TGF β treatment after three days. a) Representative flow cytometry histograms displaying Ki-67 expression in mock, dnTβRII, and TGF-βRII CAR T cells after three days of incubation with or without 10 ng/mL TGF-β1. The percentage of Ki-67-positive cells is indicated for each condition. b) Quantification of Ki-67-positive cells in mock, dnTβRII, and TGF-βRII CAR T cells with or without 10 ng/mL TGF-β1 exposure. Statistical analysis was performed using a two-way ANOVA test. c) Effect of different concentrations of TGF-β1 (0, 5, and 10 ng/mL) on Ki-67 expression in TGF-βRII CAR T cells. Statistical analysis was performed using a one-way ANOVA test. Statistical significance is indicated by *p*-values (***P < 0.001, ****P< 0.0001). Data represent the mean of technical triplicates ± SD. Ki-67 was measured by PE-conjugated anti-human Ki-67 antibody

 The proliferative capacity of TGF-βRII CAR T cells in response to TGF-β was evaluated using Ki-67 expression as a marker of cell division after stimulation with 10 ng/mL of TGF-β, as shown in Figure 4a. Preliminary analysis of proliferation assays indicated that mock and dnTβRII T cells did not proliferate in response to TGF-β (Figure 4b). The Ki-67 expression was notably lower in TGF-βRII CAR T cells compared to those of control groups (86.1% ± 0.3 mock and 85.9% ± 0.1 dnTβRII) when no TGF-β was added (*P*= 0.0004). However, the TGF-βRII CAR T cells demonstrated a significantly higher Ki-67 in the presence of 10 ng/mL TGF-β (96% ± 0.45) compared to the baseline level (79.7% ± 3.2, *P* < 0.0001) (Figure 4b). Moreover, the cells exhibited 92% ± 1.2 Ki-67 expression when exposed to 5 ng/mL TGF-β, which was significantly higher than the baseline level (*P* = 0.0002, Figure 4c), indicating that the TGF-βRII CAR construct can respond and proliferate even at low TGF-β concentrations.

 These results demonstrate that the TGF-βRII CAR T cell construct confers a significant proliferative advantage, enabling T cells to proliferate in the presence of TGF-β, a property not observed in mock-transduced or dnTβRII T cells.

###  TGF-β enhanced the production of pro-inflammatory cytokines in TGF-βRII CAR T cells

**Figure 5:**
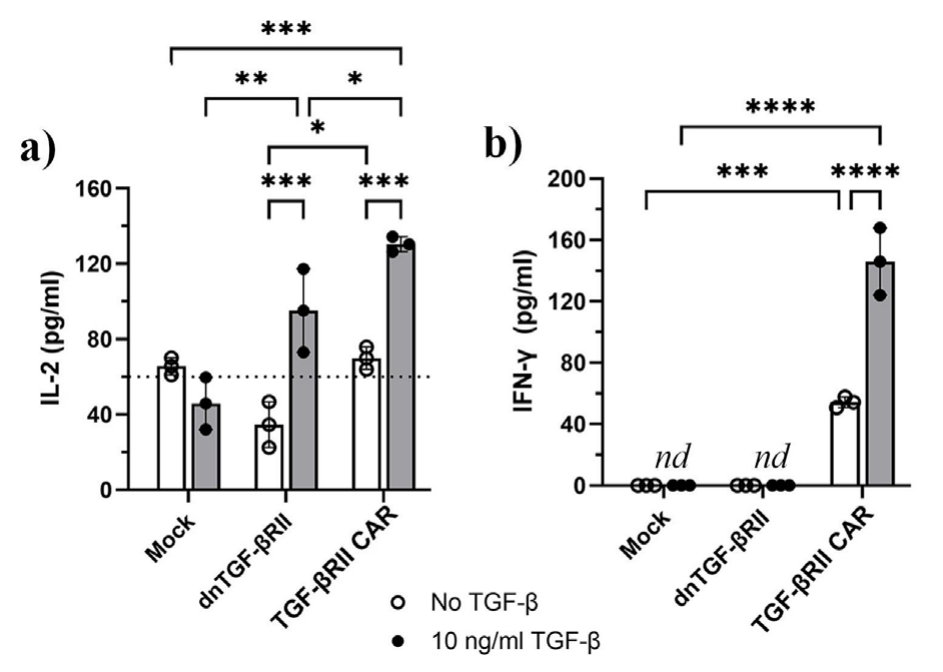
Effect of TGF-β on inducing the pro-inflammatory cytokines of IL-2 and IFN-γ by TGF-βRII CAR T cells, dnTβRII, and mock cells. a) IL-2 measurement of TGF-βRII CAR T cells, dnTβRII, and mock T cells after 48 h b) IFN-γ production by TGF-βRII CAR T cells, dnTβRII, and mock T cells after 48 h. Statistical significance is indicated by *p*-values (*P < 0.05; **P < 0.01; ***P < 0.001; ****P < 0.0001). Data shown are the representatives of three technical replications. Data are based on mean ± SD. Statistical analyses were performed using a two-way ANOVA test. IFN-γ and IL-2 secreted into the culture supernatants were measured by ELISA. Mock and dnTβRII T cells served as controls. The cells were incubated with or without 10 ng/mL TGF-β. nd: non detectable. These findings indicated that the TGF-βRII CAR T cells produced IL-2 and IFN-γ in response to TGF-β, whereas dnTβRII T cells produced only IL-2, and mock T cells showed no response upon TGF-β exposure

 The production of IL-2 and IFN-γ by TGF-βRII CAR T cells was assessed in response to varying concentrations of TGF-β (0, 5, and 10 ng/mL) and compared to control groups consisting of mock and dnTβRII T cells. As shown in Figure 5a, the production of IL-2 was significantly elevated in both dnTβRII and TGF-βRII CAR T cells following stimulation with 10 ng/mL TGF-β compared to baseline levels. Specifically, dnTβRII cells produced IL-2 at a concentration of 95 pg/mL ± 22, which was significantly higher than the baseline level of 34.6 pg/mL ± 12 (*P* = 0.0006). Similarly, TGF-βRII CAR cells showed a marked increase in IL-2 production, reaching 110 pg/mL ± 10.7 (*P* = 0.0013) and 130 pg/mL ± 4 (*P* = 0.0001) upon stimulation with 5 ng/mL and 10 ng/mL TGF-β, respectively, compared to a baseline level of 69.8 pg/mL ± 5.9 (Figures 5a and S2a). In contrast, mock cells did not exhibit a significant change in IL-2 secretion, remaining consistent at 65.5 pg/mL ± 4.5 and 45.8 pg/mL ± 13.8with or without 10 ng/mL TGF-β, respectively. Although the baseline IL-2 production levels of mock and the TGF-βRII CAR T cells were similar, dnTβRII T cells showed slightly lower levels than TGF-βRII CAR T cells (*P =*0.038). Moreover, TGF-βRII CAR T cells exhibited significantly higher IL-2 production compared to both dnTGF-βII (*p*= 0.038) and mock T cells (*P* = 0.0003) at 10 ng/mL TGF-β. Likewise, dnTGF-βII T cells produced higher IL-2 levels than mock cells (*P* = 0.0036).

 Regardless of the presence or absence of TGF-β, IFN-γ production was negligible in both mock and dnTβRII T cells (Figure 5b). In contrast, TGF-βRII CAR T cells showed a baseline IFN-γ production of 54.2 pg/mL ± 3.4 without TGF-β, which rose to 146 pg/mL ± 21.9 upon exposure to 10 ng/mL TGF-β (*P*< 0.0001). Additionally, treatment with 5 ng/mL TGF-β raised IFN-γ levels in TGF-βRII CAR T cells to 94.3 pg/mL ± 10.2 (*P* = 0.0308, Figures S2b).

## Discussion

 In this study, we aimed to develop and evaluate the effector functions of a novel TGF-βRII CAR T cell engineered to overcome the inhibitory effects of TGF-β, a cytokine known for its immunosuppressive role in the TME. We designed a series of experiments using Jurkat cells as an effective preliminary model for evaluating the engineered T cell responses to TGF-β. Although Jurkat cells are a tumor cell line and TGF-β can act as a tumor growth factor, using Jurkat cells expressing a dominant-negative TGF-β receptor II (dnTβRII) as a control enables us to separate the tumor-promoting effects of TGF-β from its specific role in activating the TGF-βRII CAR. Since these dnTβRII cells do not respond to TGF-β signaling exhibit TGF-β-mediated signaling, any increase in proliferation, activation, or cytokine production observed in the TGF-βRII CAR T cells can be attributed to CAR-mediated responses rather than TGF-β’s broader tumor-promoting effects. This comparison underscores the robustness of our approach by confirming that the observed responses are specifically driven by CAR activation through TGF-β.

 We incorporated native TGF-β receptor II in the CAR construct to recognize TGF-β, replacing the single-chain variable fragments (scFvs) typically used for antigen recognition. Traditional CAR designs relying on scFvs often face limitations such as immunogenicity, instability, and poor *in vivo* persistence, which can compromise their therapeutic efficacy.^31^ scFvs are prone to misfolding or aggregation, whereas native receptors like TβRII provide greater stability, reduced immunogenicity, and a lower risk of immune rejection, thereby enhancing the safety profile of CAR T cells.^32^ Additionally, natural receptors are better suited to adapt to the physiological conditions of the TME, such as hypoxia and cytokine gradients. By leveraging the native TGF-β receptor, our approach offers promising potential to enhance and holds promise for improving the efficacy of CAR T cells in solid tumors, which are often characterized by immunosuppressive microenvironments.

 We selected CD28 over 4-1BB because CD28 has been shown to induce faster and stronger responses, provide greater resistance to TGF-β-mediated inhibition of T cell proliferation, and consequently result in more potent effector activity.^28^ Additionally, CD28-CD3ζ CAR T cells sustain significantly higher steady-state levels of IL-2 and IFN-γ.^28,29^ The longevity and persistence of our CAR T cells, as well as their memory-like responses, remain to be determined.

 The results of this study highlight the effectiveness of the novel TGF-βRII CAR T cells in overcoming the inhibitory effects of TGF-β in the TME. By integrating the extracellular domain of TGF-β receptor II with CD28 costimulatory and CD3ζ signaling domains, these CAR T cells not only resist TGF-β-mediated suppression but also exploit its presence to drive activation and proliferation.

 The dose-response analysis of TGF-βRII CAR T cells revealed that these cells are highly sensitive to TGF-β, responding effectively to low levels (5 ng/mL), which could be advantageous in tumors with low to moderate TGF-β expression. The observed plateau at higher TGF-β concentrations (50 ng/mL) may result from receptor saturation or regulatory feedback mechanisms, potentially reducing the risk of excessive activation and cytokine release syndrome. At this concentration, the majority of available TGF-β CAR receptors are maximally engaged, and further increases in ligand availability no longer enhance downstream activation. This phenomenon reflects a well-characterized biological principle wherein receptor occupancy reaches a threshold beyond which no additional signaling gain occurs, regardless of ligand abundance. Moreover, intracellular feedback mechanisms, including residual endogenous TGF-β signaling, contribute to inhibitory signaling, potentially counteracting or dampening the activation signals induced by the CAR construct.

 The sensitivity of TGF-βRII CAR T cells was confirmed by the cytokine and proliferation assays, in which IL-2, IFN-γ, and proliferation were markedly increased at low TGF-β levels compared to the baseline level (Figure 2c, Figures S2). These findings are in line with results of TGF-β CAR T cells (anti-TGF-β scFv/IgG4 hinge/CD28/CD3z) that were manufactured using PBMCs.^33^ However, IFN-γ production in our TGF-βRII CAR T cells was much less than PBMC-derived TGF-β CAR T cells upon TGF-β exposure. This difference may be attributed to the greater affinity of the scFv and the presence of a hinge in PBMC-derived TGF-β CAR T cells, which provides a stronger interaction with TGF-β and potentially enhances downstream signaling and cytokine production. The elevated CD69 expression on our TGF-βRII CAR T cells, as well as the increased IL-2 and IFN-γ secretion upon TGF-β stimulation, compared to dnTβRII T cells, highlights the functional capability of our CAR to mediate T cell activation through CD28-CD3ζ. In CAR constructs with fused CD28-CD3ζ domains, the fusion recruits the Src family kinase Lck, which phosphorylates the Immunoreceptor Tyrosine-based Activation Motifs (ITAMs) in CD3ζ. This phosphorylation event amplifies downstream signaling cascades, including the activation of ERK (Extracellular signal-Regulated Kinase), which promotes T cell activation, proliferation, and effector function.^34,35^ Moreover, CD28 signaling in CAR T cells activates pathways such as NF-κB, PI3K/AKT, and mTOR, which promote T cell survival, proliferation, and cytokine production.^33,36^ Together, these data underscore how CD28-CD3ζ-mediated activation enables selective responsiveness to TGF-β in the TME without triggering excessive activation at higher ligand concentrations.

 The ability of our TGF-βRII CAR T cells to sustain high proliferation rates and promote pro-inflammatory cytokine production in the presence of TGF-β is noteworthy. Preclinical and clinical studies have shown that TGF-β reduces the efficacy of CAR T cell therapy in solid tumors and hematological malignancies by suppressing T cell proliferation, cytokine production, and cytolytic activity.^2,17,21,37,38^ In contrast, our TGF-βRII CAR design successfully overcomes this limitation, a feature that is not observed in the designed dnTβRII T cells. Moreover, cytokine assays demonstrated that the designed TGF-βRII CAR T cells have superior advantages over dnTβRII T cells. One of the most important properties of the TGF-βRII CAR T cells that is not observed in dnTβRII T cells is the ability of IFN-γ production upon TGF-β exposure. The increased secretion of IFN-γ by stimulated TGF-βRII CAR T cells suggests their ability to promote a pro-inflammatory microenvironment that supports anti-tumor immunity. This finding is particularly significant in the context of overcoming TGF-β-mediated immunosuppression, as IFN-γ can counteract the immunosuppressive effects of TGF-β and enhance the cytotoxic activity of T cells against tumor cells.^39^ Interestingly, we observed an increase in IL-2 production by dnTβRII T cells following TGF-β exposure, while other activation markers, including CD69 expression, proliferation and IFN-γ production, remained unchanged. These findings indicate that although dnTβRII T cells can partially evade TGF-β-mediated inhibition via producing IL-2, they may be unable to fully activate key anti-tumor functions, limiting their therapeutic efficacy. Consistently, Noh et al^40^ demonstrated that CD19CAR T cells co-expressing a TGF-β/IL-7 chimeric switch receptor exhibited enhanced cytotoxicity and improved overall survival compared to CD19CAR T cells co-expressing dnTβRII under TGF-β exposure, further emphasizing the need for strategies that not only resist TGF-β suppression but also enhance T cell activation and functionality. It is worth noting that several studies have used the CAR T cells co-expressing dnTβRII to improve anti-tumor activity of CAR T cells in TGF-β-rich TMEs.^18,20,41^ However, in TME with high TGF-β concentrations, the CAR T cells co-expressing dnTβRII may experience incomplete neutralization of TGF-β suppression, overpowering the decoy receptor and diminishing their anti-tumor efficacy.

 Our results indicated that the TGF-βRII CAR T cells designed in this study exhibited robust activation and proliferation in response to TGF-β while maintaining moderate levels of IFN-γ and IL-2, both of which are crucial for anti-tumor immunity. Importantly, the moderate cytokine profile of these cells offers a therapeutic advantage by minimizing toxicity, making them particularly suitable for solid tumors with high TGF-β levels. On the other hand, the ability of the TGF-βRII CAR T cells to proliferate upon TGF-β stimulation could lead to sustained presence and activity of CAR T cells, compensating for the lower cytokine production. In solid tumors, where active TGF-β is abundant, excessive pro-inflammatory cytokine production might not be necessary for improved efficacy of CAR T cells. Moderate cytokine levels may reduce the risk of tumor-promoting inflammation that can arise when excessive cytokines recruit immunosuppressive cells, such as regulatory T cells or myeloid-derived suppressor cells.^42-44^ Furthermore, the lower cytokine production permits administration of higher doses or extended treatment durations without increasing the risk of systemic side effects. Another advantage of these TGF-βRII CAR T cells is using them alongside immune checkpoint inhibitors, other CAR T cells, or the design of dual CAR T cells to synergistically enhance anti-tumor activity without overwhelming cytokine-driven toxicities. Studies have shown that combining TGF-β CAR T (CD4 + ) cells with either CD20 CAR T cells or NY-ESO-1 TCR T (CD8 + ) cells enhances the cytotoxicity of neighboring T cells in the presence of TGF-β.^22^ On the other hand, TGF- β can minimize a patient’s immune response to immune blockades such as PD-L1 antibodies by excluding T cells from the TME.^45^ Therefore, integrating TGF-βRII CAR T cells with PD-L1 blockade may overcome TGF-β-mediated immunosuppression and promote therapeutic efficacy.

 Jurkat cells are an ideal model cell line for early-phase mechanistic studies due to their ease of culture and transfection, cell population consistency, and uniform response to stimuli. The proof-of-concept demonstration of our TGF-βRII CAR activity in Jurkat cells establishes a foundational understanding of receptor expression, activation, and responsiveness to TGF-β. However, primary T cells differ from Jurkat cells in several aspects, including signaling thresholds, exhaustion, cytokine production, and killing capacity. Future studies will evaluate the performance of this TGF-βRII CAR in engineered primary T cells to confirm its efficacy in a clinically relevant setting. Additionally, we propose the adoption of a xenograft model utilizing NSG (NOD-scid) mice for subsequent investigations. These immunodeficient mice are ideal for human CAR T cell studies due to their lack of functional T, B, and NK cells, allowing efficient engraftment of human tumor lines and immune cells without xenogeneic rejection. The absence of murine IL-2 in NOD-scid IL-2Rγnull mice minimizes interference with human cytokine signaling, which makes these mice particularly suitable for evaluating TGF-β–responsive CAR T cells.

 Given these promising results, our TGF-βRII CAR T cells offer a versatile platform for addressing the immunosuppressive challenges posed by TGF-β in the TME. Future studies should focus on validating these findings in primary T cells and *in vivo* models, as well as exploring combinatory strategies, such as dual CAR constructs or immune checkpoint inhibitors, to enhance therapeutic outcomes. Altogether, this study highlights the potential of TGF-βRII CAR T cells as a next-generation immunotherapeutic strategy for treating TGF-β-rich solid tumors.

## Conclusion

 This study provides a TGF-βRII CAR T cell platform using a native receptor-based design that converts inhibitory TGF-β into an activating signal. Unlike traditional approaches, our CAR T cells show dual resistance to TGF-β suppression and exploitation of its presence for enhanced proliferation and controlled cytokine release. Overall, our study advances the field of adoptive T cell therapy by introducing a next-generation CAR design that turns a key immunosuppressive factor into a therapeutic advantage, offering an adaptable platform for circumventing the challenges of solid tumor immunotherapy.

## Competing Interests

 The authors declare no competing financial or non-financial interests related to the work presented in this manuscript. However, Authors acknowledge funding from Pasteur Institute of Iran, which supported a part of this research.

## Ethical Approval

 The use of human-derived samples in this study was approved by the Ethics Committee of Pasteur Institute of Iran, with ethical code IR.PII.REC.1400.031.

## Supplementary Files

Supplementary file 1 contains Figures S1 and S2.
