# Supplementary material for: A Newly Developed TGF-Β-Responsive CAR T Cell for Enhanced Proliferation and Cytokine Secretion
Source: Adv Pharm Bull. 2025 Aug 30;15(3):646–56. doi: 10.34172/apb.025.45483 (PMC12703402; doi:10.34172/apb.025.45483)
Supplement: Supplementary file 1 — contains Figures S1 and S2. [file apb-15-646-s001.pdf]

*Adv Pharm Bull*. 2025;15(3):646-S1
doi: 10.34172/apb.025.45483

https://apb.tbzmed.ac.ir

## Supplementary file 1

### Figure S1. Transfection and Transduction rate of mock T cells.

**a)** Fluorescence microscopy photograph of HEK-293 T cell transfected with mock vector. **b)** Flow cytometry analysis of transfection rate of HEK-293 T cells with mock vector after three days. The percentage of GFP-positive cells indicating successful transfection. **c)** Flow cytometry analysis of the transduction rate of mock T cells after three days. **d)** Transduction rate of mock T cells two weeks after puromycin selection**.** Controls cells (gray); transfected/or transduced cells (green).

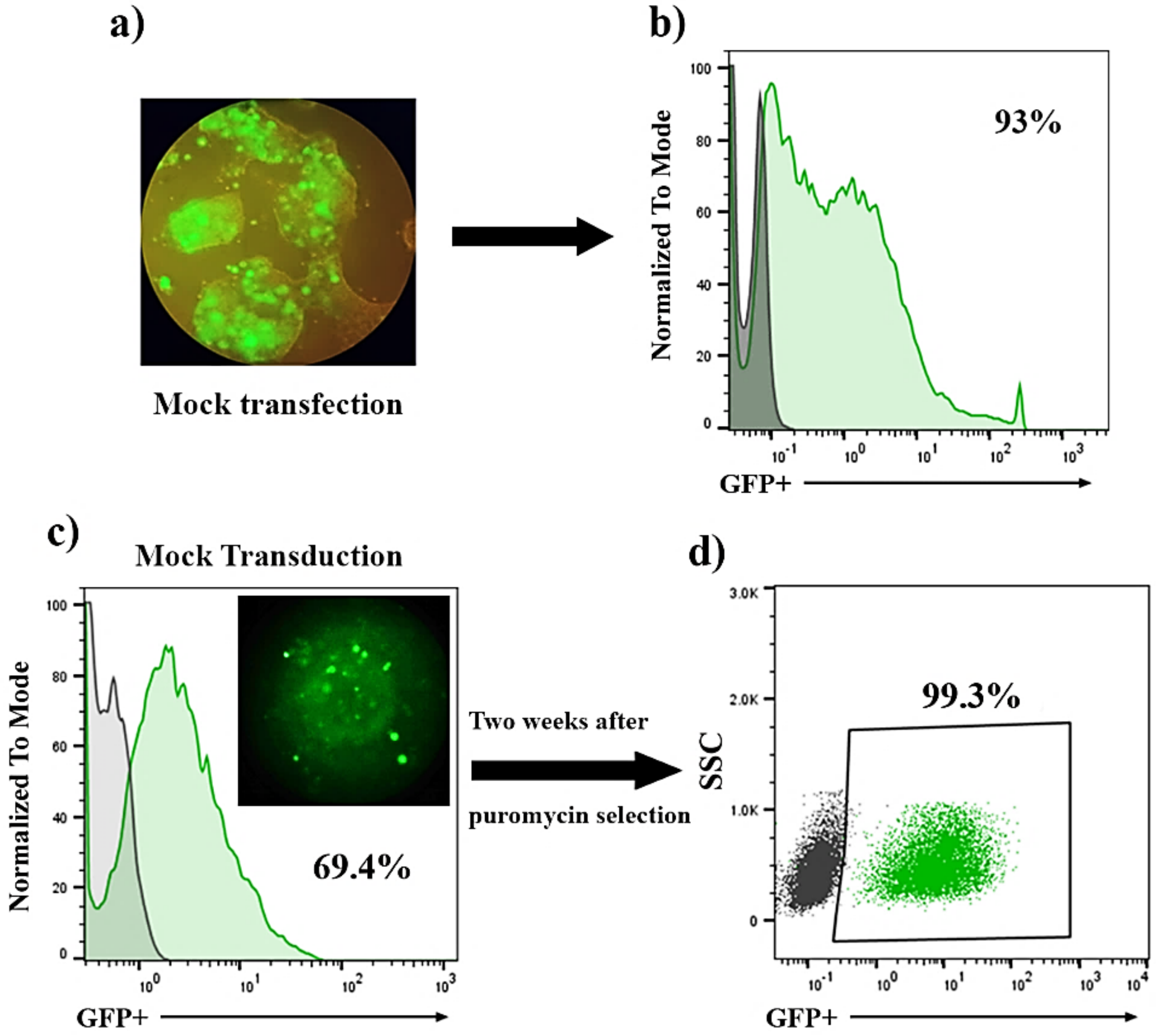


**Figure S2: IL2 and IFN-γ cytokine analysis of TGFbRII CAR T cells in response to 0, 5 and 10 ng/ml TGFb**.

**a)** IL-2 and b) IFN-γ production by TGF-βRII CAR T cells after 48h. Statistical significance is indicated by p-values (*$p < 0.05$; **$p < 0.01$; ***$p < 0.001$; ****$p < 0.0001$). Data shown are the representatives of three technical replications. data are based on mean ± SD. IL-2 and IFN-γ secreted into the culture supernatants were measured by ELISA.

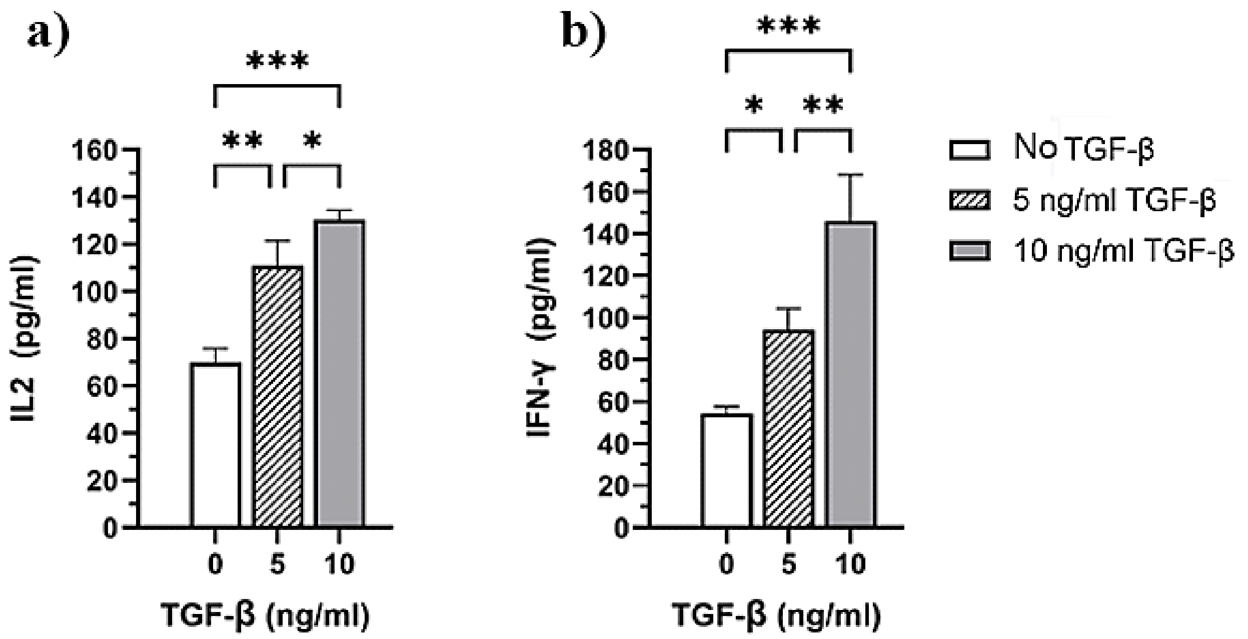
a)
***
** *
160
140
120
100
80
60
40
20
0
IL2 (pg/ml)
0 5 10
TGF-β (ng/ml)
b)
***
* **
180
160
140
120
100
80
60
40
20
0
IFN-γ (pg/ml)
0 5 10
TGF-β (ng/ml)
No TGF-β
5 ng/ml TGF-β
10 ng/ml TGF-β
